# MPDA: Microarray pooled DNA analyzer

**DOI:** 10.1186/1471-2105-9-196

**Published:** 2008-04-15

**Authors:** Hsin-Chou Yang, Mei-Chu Huang, Ling-Hui Li, Chien-Hsing Lin, Alice LT Yu, Mitchell B Diccianni, Jer-Yuarn Wu, Yuan-Tsong Chen, Cathy SJ Fann

**Affiliations:** 1 Institute of Statistical Science, Academia Sinica, Taipei, Taiwan; 2 Institute of Biomedical Sciences, Academia Sinica, Taipei, Taiwan; 3 Department of Life Sciences and Institute of Genome Sciences, Yang-Ming University, Taipei, Taiwan; 4 Genomics Research Center, Academia Sinica, Taipei, Taiwan; 5 Department of Pediatrics/Hematology Oncology, University of California, San Diego, USA

## Abstract

**Background:**

Microarray-based pooled DNA experiments that combine the merits of DNA pooling and gene chip technology constitute a pivotal advance in biotechnology. This new technique uses pooled DNA, thereby reducing costs associated with the typing of DNA from numerous individuals. Moreover, use of an oligonucleotide gene chip reduces costs related to processing various DNA segments (e.g., primers, reagents). Thus, the technique provides an overall cost-effective solution for large-scale genomic/genetic research. However, few publicly shared tools are available to systematically analyze the rapidly accumulating volume of whole-genome pooled DNA data.

**Results:**

We propose a generalized concept of pooled DNA and present a user-friendly tool named Microarray Pooled DNA Analyzer (MPDA) that we developed to analyze hybridization intensity data from microarray-based pooled DNA experiments. MPDA enables whole-genome DNA preferential amplification/hybridization analysis, allele frequency estimation, association mapping, allelic imbalance detection, and permits integration with shared data resources online. Graphic and numerical outputs from MPDA support global and detailed inspection of large amounts of genomic data. Four whole-genome data analyses are used to illustrate the major functionalities of MPDA. The first analysis shows that MPDA can characterize genomic patterns of preferential amplification/hybridization and provide calibration information for pooled DNA data analysis. The second analysis demonstrates that MPDA can accurately estimate allele frequencies. The third analysis indicates that MPDA is cost-effective and reliable for association mapping. The final analysis shows that MPDA can identify regions of chromosomal aberration in cancer without paired-normal tissue.

**Conclusion:**

MPDA, the software that integrates pooled DNA association analysis and allelic imbalance analysis, provides a convenient analysis system for extensive whole-genome pooled DNA data analysis. The software, user manual and illustrated examples are freely available online at the MPDA website listed in the Availability and requirements section.

## Background

Since the pioneering work of Arnheim *et al*. in 1985 [1], the analysis of pooled DNA samples has undergone extensive development over the past two decades [2,3]. The main applications of pooled DNA techniques in genomic/genetic studies include association mapping [4,5], polymorphism identification/validation [6,7], genetic diversity [8,9] and mutation detection [10,11]. The millennium revolution of the pooled DNA technique was its integration with microarrays [12], and the performance of which has been examined broadly [13-23]. This new-generation biotechnique significantly decreases the cost of large-scale genomic/genetic studies; for example, costs due to typing numerous DNA samples are reduced by pooling genomic DNA, and expenses related to primers and assay kits are reduced by using microarray genotyping. Therefore, microarray-based pooled DNA provides a cost-saving and valuable avenue for deciphering the mysteries of the human genome.

**Figure 1:**
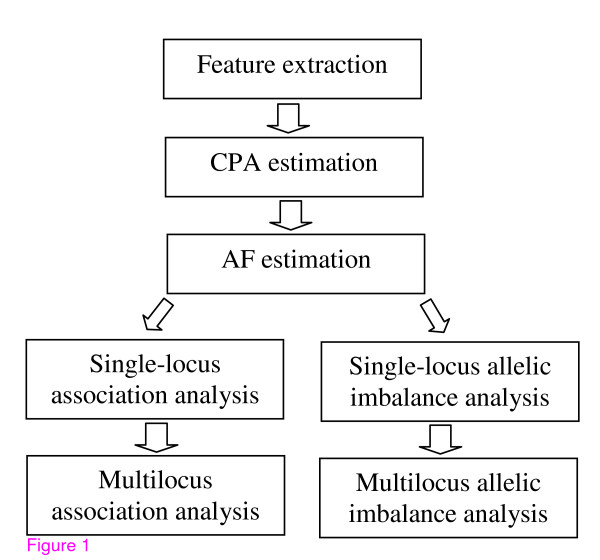
The integrated system of microarray pooled DNA analysis, MPDA.

Analysis of high-density genome-wide pooled DNA data involves a series of sophisticated procedures that require simultaneous and extensive data processing, statistical estimation and hypothesis testing. The data attributes/structures become more complicated and the computational complexity increases significantly when compared to a candidate-region or low-resolution genetic analysis. The urgent demand for efficient, publicly available software has motivated us to develop the shared software, Microarray Pooled DNA Analyzer (MPDA), which enables elaborate genome-wide pooled DNA analysis. The major functions of MPDA include data processing (feature extraction and quality evaluation), statistical estimation (whole-genome estimations of the coefficient of preferential amplification/hybridization [CPA] and allele frequency [AF]), and gene mapping (whole-genome single-locus/multilocus association analysis and single-locus/multilocus allelic imbalance analysis). Graphical and numerical outputs provide for global and detailed inspection of the human genome. Figure 1 presents the analysis framework of MPDA.

MPDA implements association analysis [24-27] and allelic imbalance analysis [28-32] based on a generalized concept of pooled DNA, of which there are two types in this study. The first is a "population-level (artificial)" DNA pool, which is constructed by mixing genomic DNA from different subjects. This pool is formed by laboratory work and reflects interindividual variations in DNA. The second type, an "individual-level (natural)" DNA pool, is contributed by a single subject. This DNA pool is formed naturally and reflects intercell variations in DNA. The artificial DNA pool concept is used to construct association analyses, whereas the natural DNA pool concept is used to develop allelic imbalance analyses.

## Implementation

### Software and interface

MPDA was developed based on MATLAB^® ^software and adapted to MS Windows^® ^98/ME/NT/2000/XP/2003. MPDA provides a user-friendly interface created using the MATLAB^® ^Graphic User Interface (see Additional files 1, 2, 3). Users can easily analyze their data by merely checking the option boxes in the MPDA interface. For users working on machines without installing MATLAB^® ^software, we also developed stand-alone executables generated via the MATLAB^® ^compiler. Moreover, two data examples reported in this paper are included in the MPDA software to demonstrate its functionalities and data input formats. The details on statistical methods and operation procedures can be found in the MPDA user manual. The software, user manual and additional data examples are available online at the MPDA website.

### Genetic typing experiments and data format

All the genotyping experiments were performed with leukocyte DNA using the Affymetrix GeneChip Human Mapping 100 K Set (Affymetrix, CA, USA) that contains 116,204 single nucleotide polymorphisms (SNPs) with a median intermarker distance of 8.5 kb and 92% genome coverage within 100 kb of a SNP [33]. Oligonucleotide probes for the SNPs were tiled on two arrays, Xba and Hind chips. Leukocyte genomic DNA was isolated using the Puregene genomic DNA purification kit (Gentra Systems, MN, USA). The DNA concentration was quantified using a NanoDrop ND-1000 Spectrophotometer (NanoDrop Technologies, DE, USA). For individual genotyping experiments, a total of 500 ng of genomic DNA with concentration at 50 ng/μl was used. For allelotyping of DNA pools, a total of 500 ng of mixed genomic DNA with an equal amount of DNA from each selected individual was used. Genotyping of each individual (also called a "natural DNA pool") or allelotyping each experimental DNA pool (also called an "artificial DNA pool") was performed according to the GeneChip Mapping Assay Protocol. The assay details can be obtained from the Affymetrix GeneChip Mapping 100 K Assay Manual [34]. For each SNP, the implanted genotyping analysis tool, GeneChip^® ^DNA Analysis Software Version 3.0, provided intensity data for the five best quartet pairs. Each quartet pair contained sense-strand and antisense-strand probe quartets, and each probe quartet consisted of perfect-match and mismatch probe cells for two different alleles.

### Feature extraction

For feature extraction from intensity data, MPDA calculated the relative allele signal (RAS) of each SNP for each subject. The average of the mismatch intensities of a pair of alleles were subtracted from the perfect match intensities to adjust background noise and yield an adjusted intensity. The adjusted intensity of an allele was divided by the sum of adjusted intensities from an allele pair to calculate a relative proportion for a strand under each quartet. Medians of the relative proportions over five quartets were calculated for sense and antisense strands respectively, and the two medians were averaged to yield the composite RAS (CRAS). The CRAS measure was a basic element of subsequent analyses.

To evaluate the quality of SNP data, MPDA calculated a discrimination signal (DS) for each SNP for each subject. For each probe pair, the sum of perfect match and mismatch intensities was divided by the difference between perfect match and mismatch intensities to yield a DS value. For each allele and probe cell, the median of DS values over five probe quartets was taken as the median DS value (MDS). A min-max criterion, which picked the minimum MDS over two probe cells for each allele and then took the maximum from the two minimums, was used to calculate the final DS (FDS) value for each SNP for each subject. The FDS can serve as an index of genotyping quality–the higher the index FDS, the better the genotyping quality of the SNP. For a good-quality SNP, an FDS of > 1.5% was suggested by the Modified Partitioning Around Medoids (MPAM) Mapping Algorithm in the Affymetrix GeneChip DNA Analysis Software User's Guide.

### Whole-genome CPA analysis

MPDA estimates the whole-genome CPA and the corresponding standard error. For any given SNP, subjects who were genotyped individually and were heterozygous with respect to the SNP were selected. Their hybridization intensities were transformed into CRAS as described in Feature Extraction. The CRAS pairs were collected and used to estimate CPA according to the arithmetic mean [35], geometric mean, and bias-correction CPA [36]. The corresponding standard error was calculated via a resampling procedure with an underlying model that the CRAS followed a beta distribution [21,37-39]. The detailed statistical formulae can also be found in Appendix A in the MPDA user manual.

MPDA provides several options of data types for the calculation of CPA. Firstly, Affymetrix users can select chromosomes of interest and provide hybridization intensity data of 100 K or 500 K to calculate CPA. Secondly, users who apply an existing CPA database can provide their own CPAs or use two CPA reference datasets provided by MPDA. Thirdly, non-Affymetrix users can input pairs of peak intensities from different platforms, such as the matrix-assisted laser desorption/ionization time-of-flight (MALDI-TOF) and the Illumina HumanHap550 Genotyping BeadChip. If users want to calculate standard error of the CPA estimate, they should enter the number of bootstrap replications into MPDA.

### Whole-genome AF estimation

MPDA estimates AFs in regards to a population-level DNA pool (Component 1 of MPDA, see Additional file 2) or an individual-level DNA pool (Component 2 of MPDA, see Additional file 3). Hybridization intensity data of DNA pools were transformed into CRAS as described in Feature Extraction. Given a SNP, CRAS were unadjusted estimates of AFs in DNA pools. To adjust preferential amplification/hybridization of alleles, the estimated CPA was multiplied by the CRAS of the second allele to amplify (suppress) the suppressed (amplified) CRAS. The new CRAS of the second allele replaced the original CRAS used in calculating the unadjusted AF to estimate the adjusted AF. The variance of the unadjusted AF estimate was calculated based on binomial sampling variation; the variance of the estimated adjusted AF included extra variation due to CPA calibration [5] and experimental error [19,40,41]. MPDA allows users to input Affymetrix-format hybridization intensity data or non-Affymetrix-format intensity pair data to estimate AFs. The detailed statistical formulae for AF estimates and their standard errors can be found in Appendix B in the MPDA user manual.

### Whole-genome association analysis

MPDA provides whole-genome single- and multi-locus association tests to screen loci susceptible to traits of interest based on population-level DNA pools (i.e., artificial DNA pools), where CPA calibration is properly incorporated into the AF estimates and association tests (Component 1 of MPDA, see Additional file 2). For single-locus association tests, differences of adjusted AF estimates of two groups are calculated, and the AF differences are divided by the corresponding standard error of the AF difference to construct the test statistics. MPDA provides two test statistics: one assumes a common CPA between two groups whereas the other does not. MPDA requires users to input the experimental error while performing the test. For multilocus association tests, the sliding-window empirical p-value test (SWEPT) statistics are used, which are composite scores of a series of p-values from single-locus association tests [42]. The single-locus p-value can come from either the MPDA single-locus association test or user-specified p-value data. Users also must select weight functions, truncation thresholds, window sizes, and three SWEPT statistics, i.e., the multiplicative-effect SWEPT, additive-effect SWEPT and minimum SWEPT. MPDA calculates an empirical p-value of the SWEPT statistic based on a Monte Carlo procedure. The number of Monte Carlo replications should be provided while conducting the test. The detailed statistical formulae for single-point and multipoint association tests can be found in Appendix C in the MPDA user manual.

### Whole-genome allelic imbalance analysis

MPDA provides an innovative whole-genome allelic imbalance analysis to identify regions of chromosomal aberration based on individual-level DNA pools (i.e., natural DNA pools), where CPA calibration is incorporated into the AF estimates and allelic imbalance analysis (Component 2 of MPDA, see Additional file 3). A SNP has alternate alleles *M *and *N*. Theoretically, for each natural DNA pool that is diploid, the AF of allele *M *should be 0, 0.5 or 1 with respect to 0, 1, or 2 copies of allele *M*. Deviations from the standards are called allelic imbalance and suggest chromosomal aberration.

We first introduce the single-locus allelic imbalance analysis. We estimated AFs for each of the healthy controls following a similar procedure for AF estimation as described in Whole-Genome AF Estimation, where the role of an artificial DNA pool was replaced by a natural DNA pool. We used the estimated AFs to establish standards representing a control population free from chromosomal aberrations, described as follows. For each SNP, we calculated the mean and prediction error of AF estimates, (${\hat{\mu }}_{uv}$, ${\hat{\sigma }}_{uv}$), under each of three genotypes {*uv*, *uv *∈ (*MM*, *MN*, *NN*)} and then constructed the corresponding prediction bands by ${\hat{\mu }}_{uv}\pm \eta \cdot {\hat{\sigma }}_{uv}$, where *η *is a critical level for determining statistical significance. Next, we estimated AFs for each patient sample following the same procedure for unaffected samples. We checked whether the estimated AF of the patient was located within one of the three prediction bands constructed from an unaffected population. If the estimated AF of a SNP was outside of the three prediction bands, then the SNP was identified as an allelic imbalance point.

Next, we introduce the multilocus allelic imbalance analysis. There are two statistics for this purpose. The first statistic is called SCORE1, which is used to detect allelic imbalance regions based on the results of the single-locus allelic imbalance analysis. Positive scores are assigned to significant SNPs (i.e., allelic imbalance SNPs) and negative scores are assigned to non-significant SNPs (i.e., non-allelic imbalance SNPs). Scores for SNP loci are accumulated from the starting SNP on each chromosome. The cumulative sum score is called SCORE1. A significantly increasing trend in SCORE1 implies that the region contains many SNPs with abnormal AFs and is a potential chromosomal aberration region. The second statistic is called SCORE2, which focuses on detecting regions that contain a chromosomal segment with a low proportion of heterozygous SNPs. All SNPs are divided into heterozygous and non-heterozygous SNPs for each patient. If the estimated AF of a SNP is located within the prediction band ${\hat{\mu }}_{MN}\pm \eta \cdot {\hat{\sigma }}_{MN}$, then the SNP is classified as a heterozygous SNP; otherwise, it is classified as a non-heterozygous SNP. Negative scores are assigned to heterozygous SNPs and positive scores are assigned to non-heterozygous SNPs. Scores for SNP loci are accumulated from the starting SNP on each chromosome. This cumulative sum score is called SCORE2. The detailed statistical formulae for single-point and multipoint allelic imbalance analyses can be found in Appendix D in the MPDA user manual.

## Results

### Whole-genome CPA analysis

Preferential amplification/hybridization, which is quantified by CPA, is a major factor interfering with the estimation of AF in pooled DNA studies. CPA has been defined as a ratio of a pair of peak intensities [36] and is related to the polymorphic nucleotide type and GC content of probes [21]. Failure to adjust preferential amplification/hybridization may result in serious bias in AF estimates thus leading to false conclusions in association mapping and allelic imbalance detection. Therefore, investigation of the pattern of whole-genome preferential amplification/hybridization and provision of cost-free CPA databases for future calibration of AF estimates in pooled DNA studies are critical [21,36,43]. We used MPDA to establish and update our CPA databases. In a new analysis, we individually genotyped 367 independent subjects recruited in our previous project "The Taiwan Han Chinese Cell and Gene Bank" [44] using the Affymetrix GeneChip Human Mapping 100 K Set. Subjects who were heterozygous with respect to SNPs from the 367 subjects were selected, and the hybridization peak intensities for both alleles from these individuals were extracted to estimate the pairs of CRAS and then estimate three CPAs of SNPs across the human genome. The corresponding standard errors for the CPA estimates were calculated based on a beta-distributed bootstrapping procedure with 1,000 replications (see Methods–Whole-genome CPA analysis).

**Figure 2:**
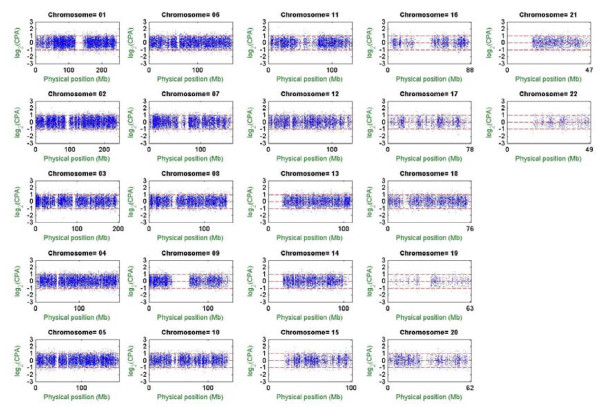
**Genome-wide distribution of CPA.** In each subfigure, the horizontal axis denotes the physical position of the SNP (scale in megabases, Mb) and the vertical axis denotes the estimated CPA in log_2 _scale. The three red lines stand for log_2_(CPA) = -1, log_2_(CPA) = 0, and log_2 _(CPA) = 1 with respect to the first allele being suppressed twofold compared with the second allele, no relative suppression/amplification, and the first allele being amplified twofold compared with the second allele.

MPDA provides both graphic and numerical outputs of CPA estimates. Figure 2 shows whole-genome scatter plots of the bias-corrected CPA estimates in log_2 _scale versus physical positions by chromosome. The gap on each chromosome represents the centromeric region; log_2_(CPA) > 0 indicates preferential amplification/hybridization of allele 1 over allele 2, and vice versa for log_2_(CPA) < 0, whereas log_2_(CPA) = 0 indicates no preference in this regard. Numerical outputs showed that whole-genome mean, median, standard deviation, minimum and maximum of the estimated log_2_(CPA) were 0.047, 0.052, 0.545, -2.570 and 2.252 respectively. The estimated CPAs play important roles in subsequent analyses, including AF estimation, association analysis and allelic imbalance analysis (see next sections). Calculations of genome-wide CPAs for the constructed Taiwanese-specific CPA database were finished within 10 hours using a PC with an Intel Pentium IV 2.4 GHz processor and 2 GB RAM. Calculations of standard error for genome-wide CPAs with 1,000 bootstrap replications were finished within 27 hours.

In addition to the Taiwanese-specific CPA database based on 367 Taiwanese samples, we also constructed another CPA database that includes the 367 Taiwanese samples as well as 42 African-Americans, 42 Caucasians, and 20 East Asians from the Human Variation Panel (Coriell Cell Repositories). The two CPA databases were then interfaced and included with the MPDA software. As the aim of MPDA is to provide a convenient tool for genome-wide genetic analysis, MPDA not only has user-friendly interfaces but also provides several options for gathering CPA information. In addition to directly using one of the two MPDA-provided databases, users can also input user-specified CPAs or carry out CPA estimation based on the supplied intensity pair data. The CPA database is applicable to different studies because of high stability across laboratories and time (see Section Discussion). However, if users would like to construct their own database, parallel computations organized by chromosomes, carried out on multiple PC's, are recommended when the sample size is large.

### Whole-genome AF estimation

**Figure 3:**
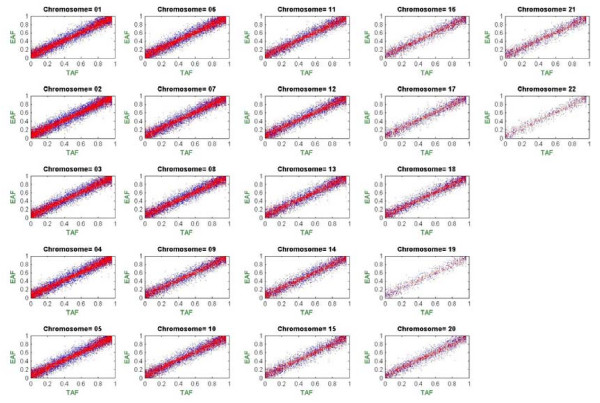
**Genome-wide distribution of the true, unadjusted and adjusted allele frequencies.** In each subfigure, the horizontal axis denotes the true allele frequency (TAF) from individual genotyping data and the vertical axis denotes the estimated allele frequency (EAF) from pooled allelotyping data, where the adjusted estimate of allele frequency is represented by red points and the unadjusted allele frequency is represented by blue points.

To illustrate whole-genome AF estimation of population-level pooled DNA data using MPDA, we carried out an allelotyping experiment based on the Affymetrix GeneChip Human Mapping 100 K Set. Equal amounts of DNA from 240 Taiwanese samples were mixed to construct an artificial DNA pool that was subjected to allelotyping using DNA chips (see Methods–Genotyping Experiments and Data Format). A set of two arrays (Xba array and Hind array) was used to assay the DNA pool. SNPs that had a call rate of < 90% in the individual genotyping data were excluded. The pairs of CRAS in the DNA pool were calculated based on the original hybridization intensity data (saved in the Example directory of MPDA). Following feature extraction, the CRAS pairs were used to calculate the unadjusted and adjusted AF estimates by incorporating the Taiwanese-specific CPA database provided in MPDA. Standard errors of the AF estimates were also calculated. The analysis was executed under Component 1 of MPDA. To examine the accuracy of the AF estimates from MPDA, the true AF was calculated by an allele counting approach using the individual genotyping data for all of the 240 study subjects involved in the artificial DNA pool (see Additional file 4). We then compared the true AF from the individual genotyping data with the unadjusted and adjusted AF estimates from the pooled DNA allelotyping data. The numerical results of the three types of AFs are presented graphically in Figure 3.

The adjusted AF estimates clearly outperformed the unadjusted estimates, verifying the importance of CPA calibration in AF estimation. Moreover, the adjusted AF highly correlated with the true AF (correlation coefficient = 0.983), demonstrating that MPDA can accurately estimate AFs in pooled DNA studies. The improvement of AF estimation was observed on every chromosome. In this example, all calculations were finished within one minute. Importantly, MPDA provides two CPA reference datasets, so users can perform preferential amplification/hybridization for AF estimation without collecting any individual genotyping data for CPA calculation, which greatly reduces the study cost and computational time for a pooled DNA study. We also performed AF estimation for individual-level pooled DNA data, and examples are presented below (see Results–Whole-Genome Allelic Imbalance Analysis).

### Whole-genome association mapping

To evaluate the performance of MPDA with regard to whole-genome association mapping based on population-level hybridization intensity data using MPDA, we performed allelotyping experiments with pool sizes of 10 and 30 individuals, respectively, using the Affymetrix GeneChip Human Mapping 100 K Set. Samples in the two artificial DNA pools were randomly drawn from the same Taiwanese population. All samples were also genotyped individually. The true AFs in the two DNA pools were calculated (see Additional file 5). SNPs that had a call rate of < 90% or were non-polymorphic in the individual genotyping data were excluded. A set of two arrays (Xba array and Hind array) was used to assay the two DNA pools, respectively. In each DNA pool, MPDA used the original hybridization intensity data (saved in the Example directory of MPDA) to calculate the adjusted AF estimate of each SNP locus by incorporating the Taiwanese-specific CPA database provided in MPDA. Pooled DNA single- and multi-locus association scans were then carried out to examine the difference in AF distribution between the two DNA pools (see Methods–Whole-Genome Association Analysis). Note that, CPA calibration should be applied to adjust for preferential amplification/hybridization, otherwise the true association may not be detected [36].

**Figure 4:**
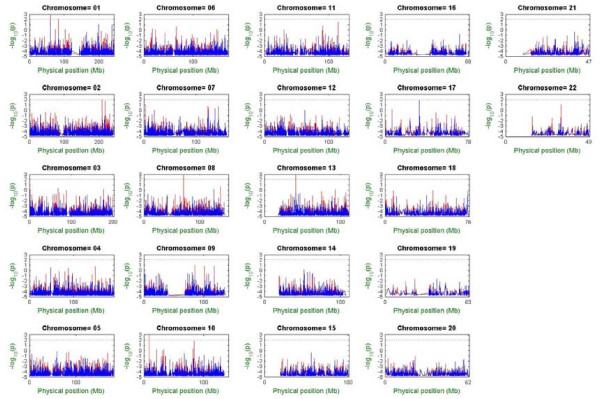
**Genome-wide single-locus association tests based on individual genotyping data and pooled allelotyping data.** In each subfigure, the horizontal axis denotes the physical position of SNPs (scale in megabases, Mb), and the vertical axis denotes the Bonferroni-type adjusted p-value (i.e., the raw p-value multiplied by the number of association tests) in a -log_10 _scale. Individual genotyping data points are in blue, and pooled allelotyping data points are in red. Each black dashed line denotes the critical line, -log_10 _(p) = 2, for the Bonferroni-type multiple tests correction.

Genome-wide single-locus association tests were carried out based on modified chi-square statistics of common CPAs, where binomial sampling error and CPA calibration error were calculated by MPDA. An experimental standard error of 0.02 was assigned; the standard error was derived from our previous experiments in which different pool sizes, multiple DNA formation, and chip replications were taken into consideration [21]. The pooled DNA single-locus p-values for SNP markers were then calculated to provide a marginal effect of a single locus. We also conducted single-locus allele-based association tests based on individual genotyping data to evaluate the performance of the population-level pooled-DNA association tests. Figure 4 presents the Bonferroni-type adjusted p-values in a -log_10 _scale across the human genome versus physical position by chromosome, where the raw (unadjusted) p-values were multiplied by the number of SNPs and then transformed to a -log_10 _scale. Because the adjusted p-values may be greater than 1, it was possible to get negative values after taking a -log_10 _transformation. Under a test size of 0.01 (i.e., the black reference line in Figure 4), results of whole-genome association mapping based on the data from pooled allelotyping and individual genotyping experiments were highly consistent except for a few loci. Note that, it is always necessary to carefully examine genotyping quality and attributes of the identified SNPs in order to reduce the possibility of false positives. In this example, the significant SNPs on chromosomes 1, 8, 10 and 13, which were identified by pooled allelotyping but not validated by individual genotyping, have either very low FDS or minor AF. This suggests that the statistical significance may be induced by measurement error. In this example, all calculations of single-locus association tests were finished within seven minutes.

We further carried out multilocus association tests. Based on the series of single-locus p-values in the order of the SNP physical positions provided by the annotation file of the Affymetrix GeneChip Human Mapping 100 K Set, we applied a multiplicative SWEPT statistic with equal weight, a p-value truncation threshold of 0.05 and a window size of 5. The final empirical p-values versus ordered windows across the human genome were calculated by chromosome, based on 10,000 Monte Carlo replications. None of the SNPs had significant AF differences (data not shown). Because the two sample pools were from the same Taiwanese population, it is not surprising that no significant SNP loci should be identified by both association tests. The running time of MPDA for multilocus association analysis depends on the number of Monte Carlo replications. In this example, all calculations of multilocus association analysis were finished within 4.5 hours.

### Whole-genome allelic imbalance analysis

**Figure 5:**
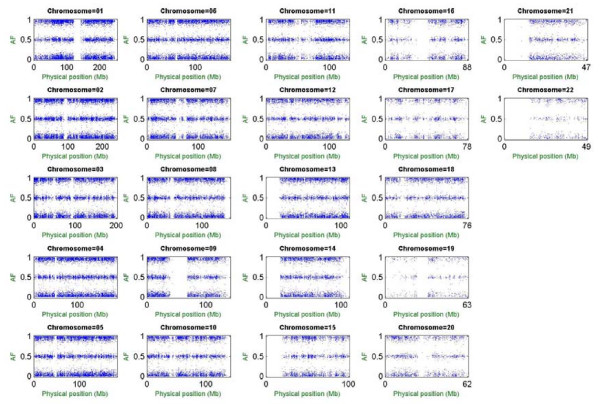
**Genome-wide distribution of AFs for a healthy control.** In each subfigure, the horizontal axis denotes the physical position of SNPs (scale in megabases, Mb), and the vertical axis denotes the estimated AFs from a healthy control.

Identification of chromosomal aberrations and allelic alterations, such as aneuploidy, gene amplification/duplication, gene deletion, and loss of heterozygosity (LOH), is an important way to identify genes involved in cancer and other diseases. For this purpose, MPDA provides an efficient allelic imbalance analysis based on individual-level hybridization intensity data. The function is illustrated by an example aiming to identify aberrant chromosomal regions associated with acute lymphoblastic leukaemia (ALL). This example involves 14 ALL patients from our previous study [45-48] and the 471 healthy controls used for CPA calculation. All samples were individually genotyped using the Affymetrix GeneChip Human Mapping 100 K Set. We carried out single- and multi-locus allelic imbalance analyses of the 14 patients (see Methods–Whole-Genome Allelic Imbalance Analysis). Whole-genome AFs for each healthy control were estimated with MPDA, and the estimated AFs were used to construct prediction bands for the genotypes *NN*, *MN *and *MM*. The whole-genome pattern of the estimated AFs for a healthy control is shown in Figure 5. Almost all AF points are located within each of the three banded regions indicating allele *M *frequencies of 0, 0.5 and 1, which correspond to the genotypes *NN*, *MN *and *MM*, respectively.

The whole-genome AFs of the 14 ALL cases were also estimated individually using MPDA. Moreover, for each patient, MPDA carried out single-locus allelic imbalance analysis to detect SNPs with signals of allelic imbalance. For multilocus allelic imbalance analysis, we calculated the cumulative sum statistic SCORE1 by assigning a value of +30 to each AI SNP and a value of -1 to each NAI SNP, and we calculated SCORE2 by assigning a value of -10 to each SNP having an AF corresponding to a heterozygous SNP and a value of +3 to each SNP having an AF corresponding to a non-heterozygous SNP. In this example, all calculations of whole-genome single-locus and multilocus allelic imbalance analyses for one patient were finished within 4.6 minutes.

**Figure 6:**
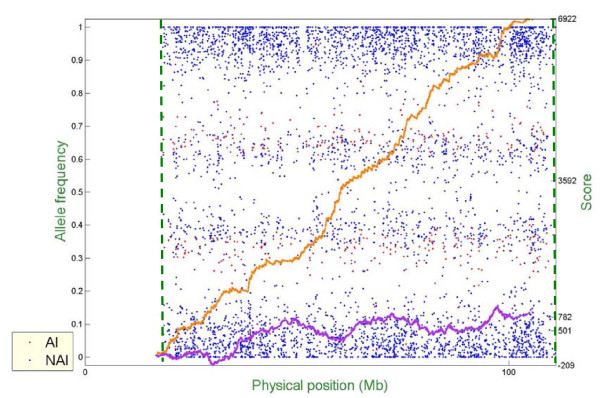
Genome-wide single- and multi-locus allelic imbalance analyses – Trisomy.

**Figure 7:**
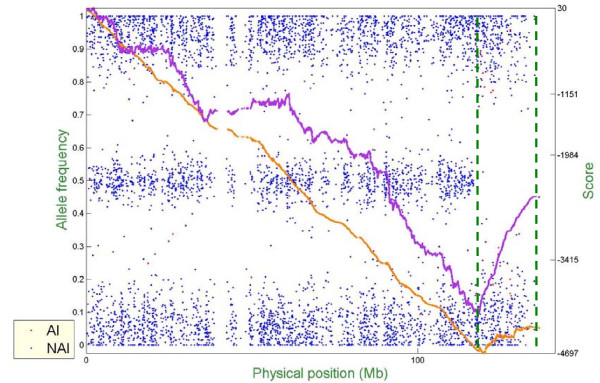
Genome-wide single- and multi-locus allelic imbalance analyses – Deletion of a microscopic chromosomal segment.

**Figure 8:**
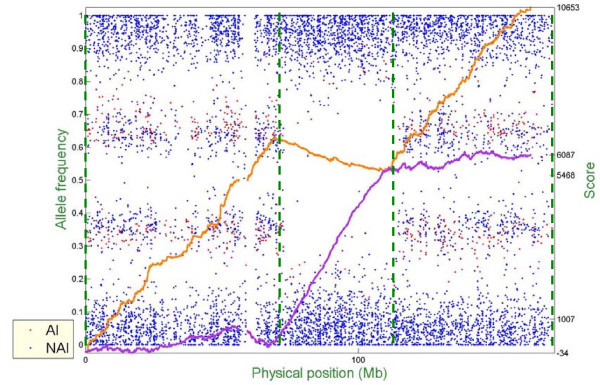
Genome-wide single- and multi-locus allelic imbalance analyses – Amplification of both ends of a chromosome but copy-neutral LOH in the middle part of the same chromosome.

**Figure 9:**
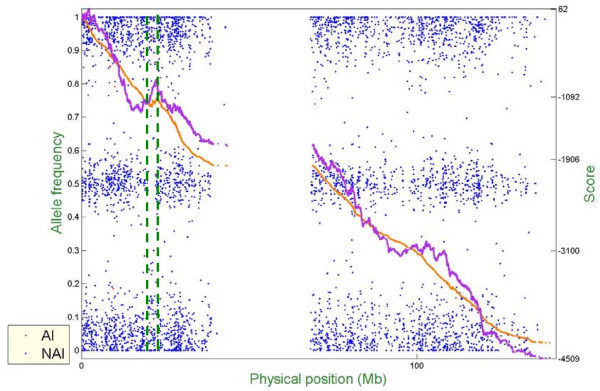
Genome-wide single- and multi-locus allelic imbalance analyses – Deletion of a submicroscopic chromosomal region.

**Figure 10:**
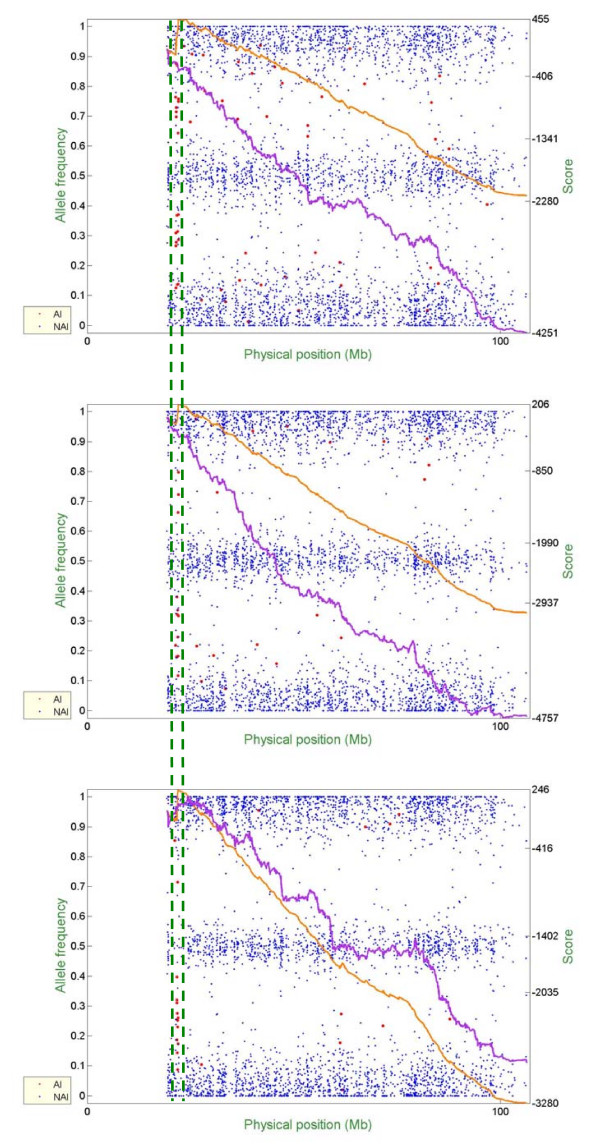
Genome-wide single- and multi-locus allelic imbalance analyses – Common deletion of the TCRα/δ locus in three ALL patients.

Figure 6 – Figure 10 show representative examples of several abnormal patterns identified by this analysis; these correspond to trisomy (Figure 6), deletion of a microscopic chromosomal segment (Figure 7), amplification of both ends of a chromosome and copy-neutral LOH in the middle of the same chromosome (Figure 8), deletion of a submicroscopic chromosomal region (Figure 9), and common deletion of the TCRα/δ locus in three ALL patients (Figure 10). In each figure, the horizontal axis denotes the physical position of SNPs (scale in megabases, Mb), the left vertical axis denotes AF, and the right vertical axis denotes the score value. Each point stands for one SNP. Non-allelic imbalance SNPs (NAI SNPs) are represented by blue points, and allelic imbalance SNPs (AI SNPs), identified by the single-locus allelic imbalance analysis, are represented by red points. The orange curve denotes the multilocus allelic imbalance cumulative sum statistic SCORE1, and the purple curve denotes the LOH cumulative sum statistic SCORE2. Results on specific chromosomes are used to illustrate different types of AI situations and the corresponding chromosomal aberrations, where dashed green lines define boundaries of aberrant regions identified by MPDA.

The relationship between allelic imbalance and DNA copy number change was validated by real-time PCR (data not shown). Importantly, allelic imbalance analysis is able to reveal biological features associated with chromosomal aberration. For example, the TCRα/δ locus on 14q11 identified by MPDA has been reported to be a frequently deleted region in ALL, partly due to somatic DNA rearrangement during T-cell differentiation. Interestingly, few SNPs on the allelic imbalance region in the three ALL patients showed normal AFs corresponding to heterozygous genotype (Figure 10), suggesting existence of two copies of the corresponding DNA segment. This implicates that DNA rearrangement of VDJ gene segments of the TCRα/δ locus rather than simple deletion, occurred in these ALL samples. Two reference databases of AF means and prediction errors derived from healthy controls are included in MPDA, thus providing a free and time-saving resource for the detection of chromosomal/allelic aberrations in a target DNA sample.

## Discussion

Compared to individual genotyping data analysis, there is very limited access to free public software for population-based pooled DNA analysis, and most of these focus on frequency estimation. For pooled DNA single-locus analysis, PoolFilter [49] estimates AF for microsatellite markers. For pooled DNA multilocus analysis, the packages LDPooled [50], EHP.R [51] and Pools2 [52] use expectation-maximization algorithms to estimate haplotype frequency. However, the complicated computations lead to the restriction of small pool size and/or small number of markers in the analysis. None of these three packages allows automatic selection of SNPs involved in the haplotype analysis or contains a multilocus association test. Although these methods have pioneered pooled DNA multilocus analysis, these limitations restrict their application to practical whole-genome pooled DNA analysis. For genome-wide microarray pooled DNA data, EMMURAS.R [20] is a collection of R-language functions that provides data pre-processing, including the calculations of discrimination score and RAS. PPC [53] is an algorithm that estimates AF for the Affymetrix GeneChip Human Mapping 10 K Array. GenePool is a recently published package [22] that provides AF estimation and association tests for microarray data. The major difference between GenePool and MPDA is that MPDA provides both data analysis for natural DNA pools as well as artificial DNA pools, but GenePool provides data analysis only for artificial DNA pools. There are other programs that provide partial analysis for pooled DNA data (e.g., the Affymetrix GDAS), but these have not been released for free public access. Our previously developed PDA [42] provides single- and multi-locus association tests by analyzing peak intensity data from matrix-assisted laser desorption/ionization time-of-flight mass spectrometry [6,54-57]. PDA also handles data from different genotyping platforms; however, it is not straightforward to use for whole-genome microarray pooled DNA data analysis even though supplementary sophisticated data processing can be handled by users themselves.

In this paper we introduce MPDA, which has a user-friendly interface and applications for studying two different types of pooled DNA. The MPDA extends the advantages and capabilities of existing software to provide an integrated analysis system for whole-genome microarray pooled DNA studies. Importantly, in addition to its functions of feature extraction, CPA estimation and AF estimation, MPDA is applicable to both population-level and individual-level DNA pools and thus is a useful approach to systematically identify disease susceptible genes by detecting marker-trait association and aberrant chromosomal regions inferred from allelic imbalance. The proposed association analysis provides a reliable whole-genome screening method. The results help to dramatically reduce the number of study SNPs involved in the follow-up confirmation study and thereby reduce costs. The proposed analysis of allelic imbalance provides complementary and auxiliary information on chromosomal abnormalities for other detection approaches, such as copy number estimation [58-62]. For example, as illustrated in Figure 8, allelic imbalance analysis can detect a genomic aberrant region with copy-neutral LOH, which can not be detected using copy number analysis. Compared to the other software for pooled DNA analysis, to our knowledge, MPDA is the first software that integrates whole-genome pooled DNA analysis and chromosomal aberration detection.

**Figure 11:**
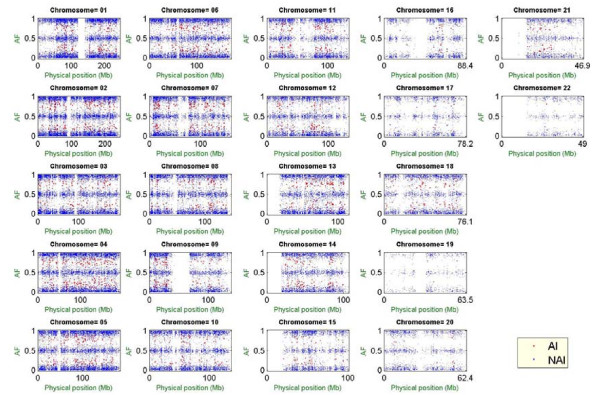
Quality control analysis for a healthy control.

Moreover, the allelic imbalance method can also be applied to monitor genotyping quality and identify copy number polymorphisms. We have applied this method to healthy controls who had been genotyped individually to check the patterns of the estimated AFs. For example, as shown in Figure 11, the whole-genome AFs of a healthy control showed an obviously abnormal pattern where a significant proportion of SNPs were located outside the three prediction bands (i.e., the red points in Figure 11) on most chromosomes. Because no identifiable disease was reported for this individual during sample collection and due to the dispersed distribution of the SNPs with abnormal AFs across the whole genome, this result strongly suggests that contamination with another genome(s) occurred during DNA preparation or genotyping procedures. In addition, our proposed allelic imbalance analysis is potentially useful to identify copy number polymorphisms, which are useful genetic markers for gene mapping and gene testing of microdeletion or microduplication disorders [62]. In addition to analyzing copy number polymorphisms using individual-level hybridization intensity data, we are also developing methods to validate/identify copy number polymorphisms based on population-level pooled DNA data. Polymorphism validation/identification using population-level pooled DNA data is cost-saving and efficient for SNPs [6,7,54] and other genetic polymorphisms [49,63-65].

Analyses in MPDA rely on the provided reference databases of CPA and prediction bands from individual genotyping data. The methods are feasible only if the reference databases are stable and applicable to analyses performed in different laboratories and conditions. Therefore, we evaluated the stability of CPA and AF based on individual genotyping data from different laboratories and time as follows. (1) Stability of CPA and AF across laboratories: we calculated CPA based on individual genotyping data of our 367 Taiwanese samples and 90 Asian samples in the HapMap project, respectively. The Pearson correlation coefficient between the two sets of CPAs was 0.913. In addition, the two sets of CPA were applied to adjust AF in the example discussed in Section Whole-genome AF estimation. The correlation coefficient between the two sets of AF estimates was 0.995. The high correlation coefficients demonstrate the stability of CPA and AF estimation based on different reference data from different laboratories. (2) Stabilities of CPA and AF across genotyping time: we partitioned our 367 Taiwanese samples into five subsets according to their genotyping time. The five subsets contained 95, 10, 96, 90 and 76 samples, respectively; the second subset was excluded from the analysis because of the small sample size. Then we calculated CPAs of the four subsets (called S1, S2, S3 and S4) in order. Correlation coefficients of CPAs from each pair of subsets were calculated. They were Corr(S1,S2) = 0.975, Corr(S1,S3) = 0.971, Corr(S1,S4) = 0.965, Corr(S2,S3) = 0.971, Corr(S2,S4) = 0.968, and Corr(S3,S4) = 0.967. In addition, the CPAs from different subsets were applied to adjust the unadjusted AFs in the example discussed in Section Whole-genome AF estimation. The correlation coefficients between any pair of AFs were 0.999. The high correlation coefficients demonstrate the stability of CPA correction across genotyping time. We followed similar methods to investigate the stability of prediction bands, and high correlations of prediction bands across laboratories and time were observed. The results verified the stability of the MPDA-provided databases.

We also evaluated the robustness of our allelic imbalance method. Firstly, the assumption that a genome is diploid is inherent in most genotype calling algorithms [33,66,67]. Deviation from diploidy causes genotype calling errors, thereby resulting in misleading conclusions in subsequent analysis [68]. This assumption is particularly impractical for genotyping DNA from cancer cells, which usually have abnormal copy number for chromosomal segments. Our approach avoids this problem by estimating AFs for each SNP of patients without requiring genotype information. Secondly, all genotyping experiments fail to completely preclude biological noise and technical noise that are inherent in microarray data. For example, in Figure 5, the noise might have caused AFs of a few SNPs from a healthy control sample to deviate from the expected value for a normal reference population. Our method adjusts background noise during feature extraction and alleviates abnormal interference by incorporating multiple loci. Even though noise is not completely eliminated, the results from real examples show high sensitivity and specificity in our association and allelic imbalance analyses. Thirdly, although all the examples illustrated in this paper were based on the Affymetrix GeneChip Human Mapping 100 K Set, the methodology in MPDA is general and also applied to genotyping arrays with higher marker density, such as the Affymetrix GeneChip Human Mapping 500 K Set. Moreover, MPDA not only accommodates the Affymetrix gene chips but also the Illumina bead chip genotyping platform by inputting pairs of normalized image intensities. Users can follow the instruction in Section 5 of the MPDA user manual to prepare the input data.

## Conclusion

In summary, we have developed a convenient tool, MPDA, for whole-genome microarray-based pooled DNA analysis. The four authentic data examples presented in this paper demonstrate the feasibility and important applications of MPDA. The first analysis–based on genotyping data of 367 subjects–shows that MPDA can be used to identify genomic patterns of preferential amplification/hybridization and provide important calibration information for pooled DNA data analysis. The second analysis yielded a correlation coefficient of > 0.98 between adjusted AF estimates based on allelotyping data from a DNA pool and true AFs based on individual genotyping data of the samples, demonstrating that MPDA can accurately estimate AFs based on pooled DNA data. The third analysis compares the results of two types of association mapping based on pooled allelotyping as well as individual genotyping data, and the results indicate that MPDA is cost-effective and reliable for association mapping. The final analysis–based on genotyping data of 14 ALL patients–detected allelic imbalance in cancer genomes, indicating that MPDA can identify regions of chromosomal aberration in cancer and other diseases. Graphic and numerical outputs are simultaneously generated by MPDA to support global and detailed inspection of large quantities of genomic data. All these features make MPDA a useful tool for analyzing allelotyping/genotyping hybridization intensity data for various research interests.

## Availability and requirements

The MPDA software, user manual and illustrated examples can be downloaded from the MPDA website: .

**Project name: **Microarray pooled DNA analysis project

**Project home page: **

**Operating system: **MS Windows^®^

**Programming language: **MATLAB^®^

**Other requirements: **No

**License: **MPDA license

**Any restrictions to use by non-academics: **On request and citation

## List of abbreviations used

MPDA: Microarray pooled DNA analyzer; DNA: Deoxyribonucleic acid; CPA: Coefficient of preferential amplification; AF: Allele frequency; SNP: Single nucleotide polymorphism; RAS: Relative allele signal; CRAS: Composite relative allele signal; DS: Discrimination signal; MDS: Median discrimination signal; FDS: Final discrimination signal; SWEPT: Sliding-window empirical p-value test; LOH: Loss of heterozygosity; ALL: Acute lymphoblastic leukaemia; AI: Allelic imbalance; NAI: Non-allelic imbalance.

## Authors' contributions

HCY conceived the experimental designs, statistical methods, performed data analyses and prepared the manuscript. MCH programmed the software. LHL contributed to the discussion and prepared the manuscript with HCY. CHL constructed DNA pools in the allelotyping experiments. ALTY and MBD provided DNA samples for the ALL patients. LHL, JYW, YTC and CSJF provided support for the genotyping and allelotyping experiments.

## Supplementary Material

Additional file 1 Interface 1 of MPDA. The welcome interface of MPDA. Click here for file

Additional file 2 Interface 2 of MPDA. The interface to MPDA association analysis. Click here for file

Additional file 3 Interface 3 of MPDA. The interface to MPDA allelic imbalance analysis. Click here for file

Additional file 4 True AFs in one group. Results of the true AFs from individual genotyping data for the 240 study subjects involved in the artificial DNA pool. Click here for file

Additional file 5 True AFs in two groups. Results of the true AFs from individual genotyping data for the 10 and 30 study subjects involved in the two artificial DNA pools. Click here for file
