# Supplementary figures and images for: MPDA: Microarray pooled DNA analyzer
Source: BMC Bioinformatics. 2008 Apr 15;9:196. doi: 10.1186/1471-2105-9-196 (PMC2387178; doi:10.1186/1471-2105-9-196)

**Supplementary Figure 1.** Interface 1 of MPDA—Main interface

**
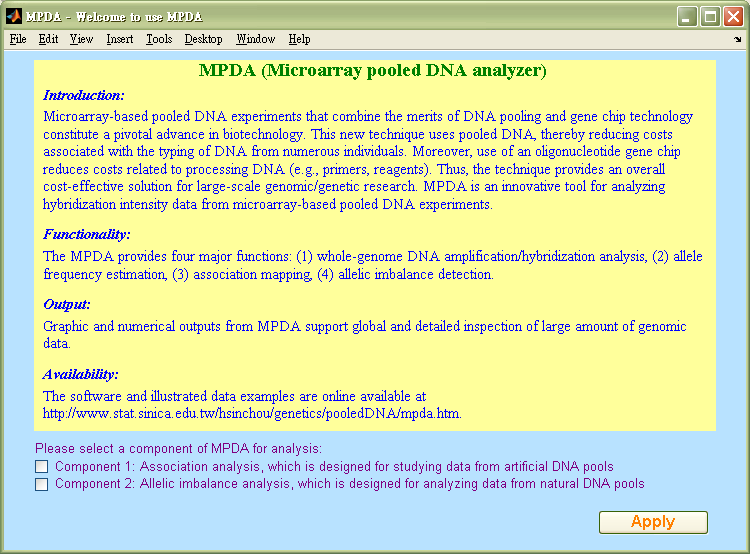
**

Supplement: Additional file 1 — Interface 1 of MPDA. The welcome interface of MPDA. [file 1471-2105-9-196-S1.doc]

**Supplementary Figure 2.** Interface 2 of MPDA—Association analysis

**
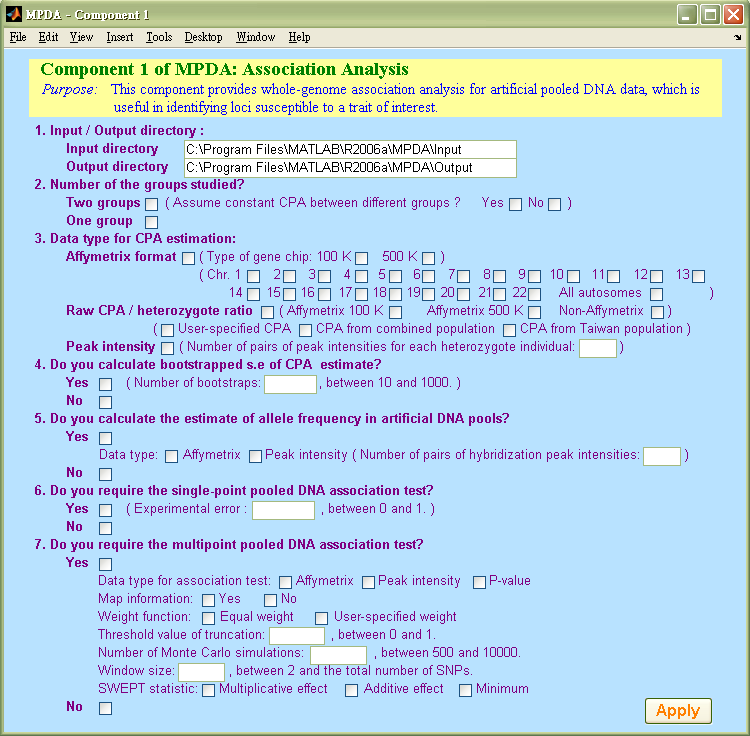
**

Supplement: Additional file 2 — Interface 2 of MPDA. The interface to MPDA association analysis. [file 1471-2105-9-196-S2.doc]

**Supplementary Figure 3.** Interface 3 of MPDA—Allelic imbalance analysis

**
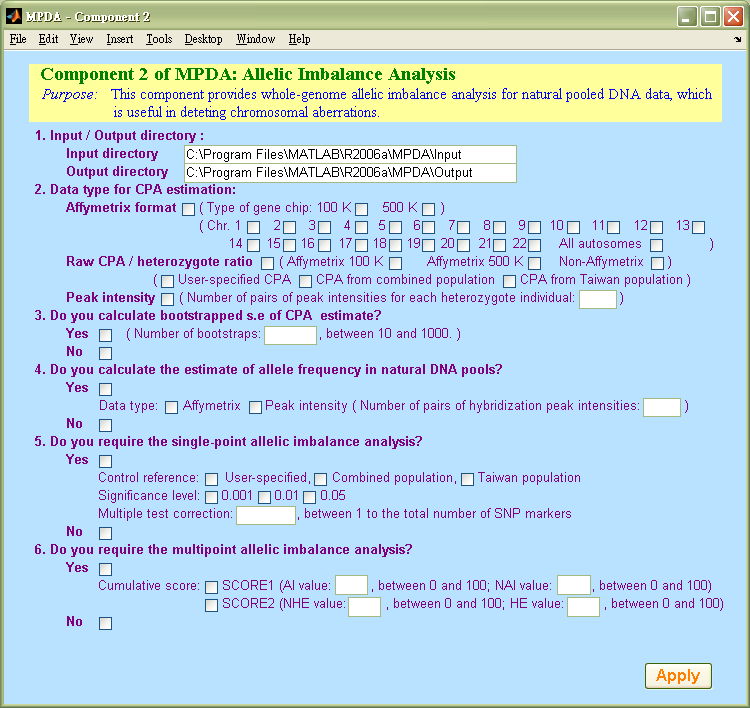
**

Supplement: Additional file 3 — Interface 3 of MPDA. The interface to MPDA allelic imbalance analysis. [file 1471-2105-9-196-S3.doc]
